# An expert botanical feature extraction technique based on phenetic features for identifying plant species

**DOI:** 10.1371/journal.pone.0191447

**Published:** 2018-02-08

**Authors:** Hoshang Kolivand, Bong Mei Fern, Mohd Shafry Mohd Rahim, Ghazali Sulong, Thar Baker, David Tully

**Affiliations:** 1 Department of Computer Science, Liverpool John Moores University, Liverpool, United Kingdom; 2 Universiti Tunku Abdul Rahman, Jalan Sungai Long, Bandar Sungai Long, Cheras, Kajang, Selangor, Malaysia; 3 Media and Games Innovation Centre of Excellence (MaGIC-X) UTM-IRDA Digital Media Centre, Institute of Human Centred, University Industry Research Laboratory (UIRL), Universiti Teknologi Malaysia UTM, Skudai, Johor, Malaysia; 4 Universiti Malaysia Terengganu, Terengganu, Malaysia; United States Department of Agriculture, UNITED STATES

## Abstract

In this paper, we present a new method to recognise the leaf type and identify plant species using phenetic parts of the leaf; lobes, apex and base detection. Most of the research in this area focuses on the popular features such as the shape, colour, vein, and texture, which consumes large amounts of computational processing and are not efficient, especially in the Acer database with a high complexity structure of the leaves. This paper is focused on phenetic parts of the leaf which increases accuracy. Detecting the local maxima and local minima are done based on Centroid Contour Distance for Every Boundary Point, using north and south region to recognise the apex and base. Digital morphology is used to measure the leaf shape and the leaf margin. Centroid Contour Gradient is presented to extract the curvature of leaf apex and base. We analyse 32 leaf images of tropical plants and evaluated with two different datasets, Flavia, and Acer. The best accuracy obtained is 94.76% and 82.6% respectively. Experimental results show the effectiveness of the proposed technique without considering the commonly used features with high computational cost.

## Introduction

One of the imperative steps to preserve and conserve the biological diversity is to automatically recognize, understand, and identify them. Conventionally, plants are classified and catalogued based on the plant taxonomy method in a manual manner using a human operator. This method relies heavily on a professional botanist, which is time consuming, tedious, cumbersome, high cost, and a potential error prone task. However, the sharply development in computer technology in recent decades provide a potential opportunity to digitize and computerize the plant identification methodology.

Based on plant taxonomy theory, a plant can be identified based on their external structure such as leaf, seed, flower, and fruit [1]. However, in this paper, only characteristics of a leaf are derived to identify the plant species.

**Fig 1:**
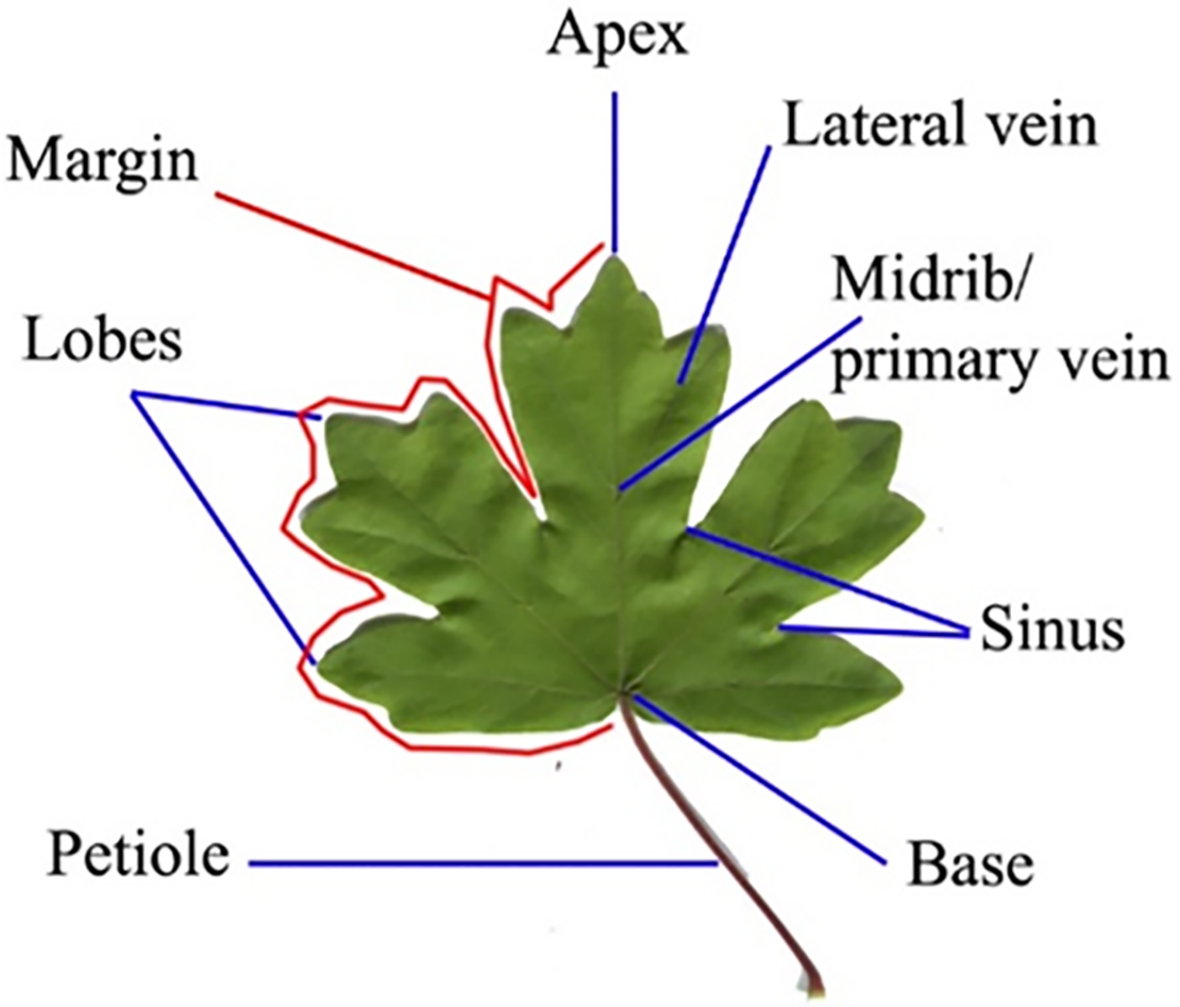
External leaf structure.

Lobes and sinus are part of the leaf margin with “large teeth”. The roundish projection part is known as lobes as shown in Fig 1. The sinus is always located between two lobes. The ratio of the distance of the teeth to the distance of margin to the midrib that exceeds 1:8 is considered as lobes and sinuses. Otherwise, it is teeth.

Leaf apex is the upper region of the leaf, which covers ¼ of the entire leaf. This region is located opposite the petiole. Some botanists’ call this region the leaf tip. Leaf apex is divided into a few types by botanists; namely acuminate, cuspidate, acute, rounded, obtuse, and truncate.

The apex shape depends on the curvature pattern of leaf apex. There is no measurement for leaf apex in a botanical perspective. The botanist determined the apex shape based on the description of the curve in the leaf apex or leaf tip. However, many previous researchers used the angle of apex to describe the shape of the apex. Botanists’ does not accept this, as many different types of leaf apex share the same angle.

The base of a leaf is the lower part of the lamina, where it is attached to the petiole or stem. The base region is located at the bottom part near the petiole or stem. The shape of the leaf base is used to identify the plant species. The uniqueness of leaf base is also described in words by botanists. Fig 1 shows the external leaf structure of a leaf image.

Leaf base, which has both of its sides, gradually taper to a narrow basal and incurved or slightly incurved are called ‘*attenuate’*. For the leaf base, which is called ‘*cuneate’*, has both sides approximate to a straight line. For the leaf base called ‘*obtuse’*, both of its sides taper to a narrow wedge-shaped base. The sides of the leaf, which have a smooth arc are called ‘*rounded base*’. The leaf base called ‘*cordate*’ has a heart shape and a gently lobed base.

In this paper, we have recognised leaves based on three properties including apex, base, and margin, by considering the lobes. Although we have considered Phenetic parts of the leaf, the results are very insightful’, and can be simply extended by involving some other parts such as shape, texture, and venations [2] to get to improve accuracy.

## Related work

This section discussed the summary of related work on the leaf apex, and leaf base. However, these features are important in botanical knowledge. The popular methods to analysis the features of leaf apex and base are to compute the angle of leaf apex and base and Gradient in sub-window.

To detect the leaf apex and base, the petiole of leaf must be identified. Mouine et al. [3][4][5][6] removed the petiole by finding the smallest distance between left and right leaf edge. Pahalawatta [7] and Gouveia et al. [8] also applied the same method to remove the leaf petiole.

After removing the leaf petiole, Yahiaoui et al. [9] detect the leaf apex and base by finding the convex or indentation of leaf contour by using Local Translational Symmetry. They then improved their approaches by adding adaptive selection of the threshold to select a better region of the leaf apex and base [10][11]. After the leaf apex and base detection, Mzoughi et al. [12] used the leaf apex and base which were then described in term of shape and texture. They applied Hue Geometric and Digital Morphology to capture the shape information. Then, Fourier histogram, edge orientation, and local edge orientation histogram were used to capture the venation on the leaf apex and the leaf base respectively [2].

Hati and Sajeevan [13] calculated the angle of apex and base angle in $\frac{1}{4}th$ and $\frac{1}{10}th$. Ab Jabal et al. [14], and Arun Priya, Balasaravanan and Thanamani [15] only found the angle of leaf tip in $\frac{1}{4}\text{th}$ from leaf length regardless of its base information. Although the angle of leaf apex and base is easy, fast, and convenient to detect, this methods is unable to distinguish the tiny changes of the leaf apex and base. This is because the leaf apex and base that share the same range of angle may have different patterns. The information of angle is not enough to extract the curvature information of leaf apex and base.

Pahalawatta [7] sub-divided the leaf apex into several sub-windows and then the gradient of the leaf apex’s contour in each sub-window is computed. This method is easy to apply; however, this method is unable to detect the abrupt changes of the leaf apex and base. Besides that, this method is variant to geometrical transformation and the result may influence by the starting point.

Previous researchers, such as Hati and Sajeevan [13], Arun Priya [15], measured the angle of the leaf apex and base to make this measurement as a parameter to interpret the leaf apex. However, in the perspective of botanical knowledge, all leaf bases with the same angle are actually not the same. Botanists acknowledge the shape of the leaf base, and the margines of the leaf base, are noted to be incurved. This characteristic of the leaf is called ‘attenuate’. When the margin of the leaf base which is approximately straight call *cuneate*. From the description of botanical knowledge, the proposed method is able to introduce a new way to extract the curvature information about the base and apex shape, which is named as Centroid Contour Gradient (CCG) [16].

The states of shape for a curved leaf, which have the longest width near its apex, are classified as; *oblanceolate*, *obovate*, *widely obovate*, and *very widely obovate*. Their differences are also dependent on the ratio of length to the width.

Leaf margin is the edge or the outline of the leaf. The leaf margin can be divided into a toothed leaf or a non-toothed leaf. A non-toothed, or smooth margined leaf, is call ‘*entire’*. For the toothed leaf, the size of tooth can be divided into fine tooth or teeth. For the leaf with fine or diminutive teeth, the ratio between the distance for leaf teeth and the distance of teeth to the midrib are between 1:16 to 1:8. However if the ratio is less than 1:8, its teeth is considered as normal teeth. For those greater than 1:8, the margin is not teeth, but are lobes.

Narayan and Subbarayan [17], and Pornpanomchai [18][19], proposed a very simple measurement to interpret the leaf margin by calculating the number of ripples and the total number of pixels in ripples. The first found the average of leaf boundary, and then found the differences of it with leaf image. The weakness for this method is it is unable to distinguish the type of the leaf.

An’s [20] did the same work but the sampled leaf used is compound leaf which is different with single leaf (lobed leaf). In our research, we focus on single leaf as the database used is single leaflet. For compound leaf, there is no need to select the terminal apex as all of them are considered as the same. For lobed leaf (single leaf), there is a need to select which part is the terminal apex. Some apex have two peaks and some peaks are not considered as apex, but belongs to the margin. Some of the leaf base has two curves, but some only have one peak as their base.

Based on plant taxonomy theory, a plant can be identified based on their external structure such as leaf, seed, flower, bark, and fruit [1]. Their physical traits can be digitalized and used to distinguish among them. An [20] used the length of the leaf and rosette area to identify the plants. Jelinkova et al.[21] used the digital morphometric of bark and the shape of the leaf to distinguish the Aspen clone. The extracted physical traits can be in qualitative and quantitative characters which had been proved by Petchsri et al. [22]. However, in this paper, only characteristics of a leaf are derived to identify the plant species.

## Method

In this research, botanical features are used to detect the interest regions of the leaf part and extract the features of leaf part. A total of seven leaf features are needed to identify the species of a plant. These are leaf shape, leaf lobes and sinuses, apex, base, margin, venation, and texture. In these seven features, only texture features did not embed botanical features.

It is somewhat obvious that the region detection will need to know the outline. Then, the regions of lobes and sinuses are detected using local maxima and local minima. The regions of apex and base are located $\frac{1}{4}\;\text{th}$ and $\frac{3}{4}th$ of the whole length separately. Finally, the regions of venation are the skeleton of the leaf.

Feature extraction is a critical role in this research for leaf classification. For the features of the leaf shape, it is distinguished based on the botanical knowledge. The terminology of the features are translated into the computer language. For the features of lobes and sinuses, the numbers and the location of lobes and sinuses acted as the features of lobes and sinuses. For apex and base, the pattern of apex and base curvature is used to interpret them by using botanical features. Finally, the teeth pattern is used to represent the features of margin.

### External leaf structure detection

Each external leaf structure has its own features and characteristics. Botanists’ and taxonomists’ used the external leaf structure to distinguish their plant species. This method is still widely used as it is higher accuracy, compared to phylogenetic approaches. In the taxonomy and botany field, the study of the features of external leaf structures’ is called ‘*plant morphology*’. However, before extracting the features of external leaf structure, it is necessary to detect the regions of the external leaf structure.

#### Lobes and sinuses detection

Curvature maxima and curvature minima are used to detect the projecting parts of a leaf. These features are called ‘*lobes*’. The indented parts are called ‘*sinuses*’. Contour Centroid Distance for Every Boundary Point (CCD-EBP) is used to detect the curvature maxima and curvature minima of the leaf. CCD-EBP is also applied to differentiate the entire-edge of the leaf, and toothed classification, and further determined the shape of the leaf lamina. This method computes the distance of centre point to all boundary points. The x and y axis value of the leaf boundary are collected in a clockwise direction and put into two vectors called *BX*_*i*_ and *BY*_*i*_. The parameter *i* indicates the sequence number of boundary point in clockwise direction. The starting point of *BX*_*i*_ and *BY*_*i*_ can be any boundary point.

In Euclidean plane, the length of the line extended from the centroid point (*C*_*x*_, *C*_*y*_) to the boundary point (*BX*_*i*_, *BY*_*i*_) is measured by using the Euclidean Distance. Euclidean Distance is derived from Pythagorean Theorem as shown in Eq 1. The distance for every single boundary point and centroid point is collected in a vector and declared as *Dist*_*i*_, where *i* represents the sequence number of element in the boundary vector and it is a real number which is *i = {1*,*2*,*3*,*…*, *n}*. The parameter *n* represents the total number of boundary points.

$${Dist}_{c}=\sqrt{{\left({BX}_{i}-C_{x}\right)}^{2}+{\left({BY}_{i}-C_{y}\right)}^{2}}$$ (1)

Our method presented to conventional Contour Centroid Distance (CCD). Conventional CCD only computes the distance of the centroid point and boundary points which is located in the interval angle. For example, if the interval angle is increased by 10 degrees, only 36 (360/10) distinct boundary points are selected to find their distance from the centroid point.

**Fig 2:**
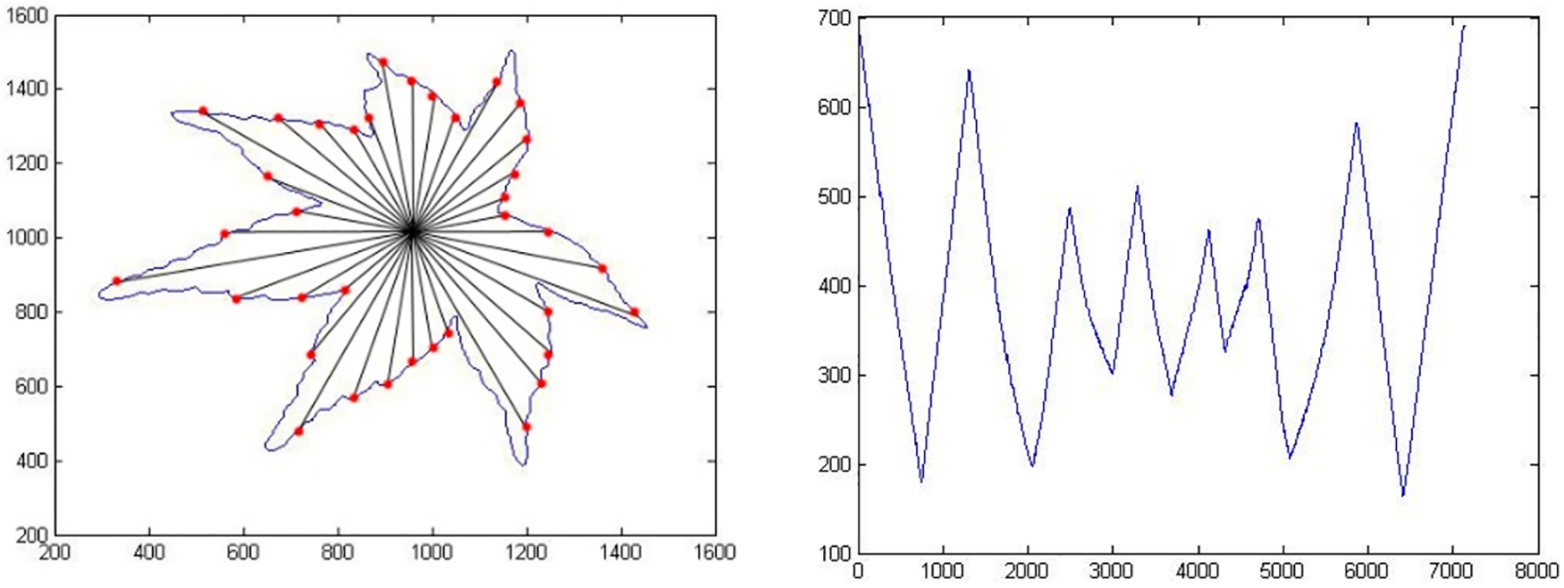
(Left): Selected boundary point by interval angle with increment of ten degrees (miss hit to local maxima and local minima point), (Right) CCD-EBP in graph.

Fig 2 (left) shows the selected boundary points, which are used to represent the shape signature by using CCD. However, they fail to hit the local maxima and local minima and are not significantly representative of the shape of the leaf lamina. It is necessary to compute every single boundary point with its centroid point to find their local maxima and local minima, and would not miss out any significant local maxima and local minima.

**Fig 3:**
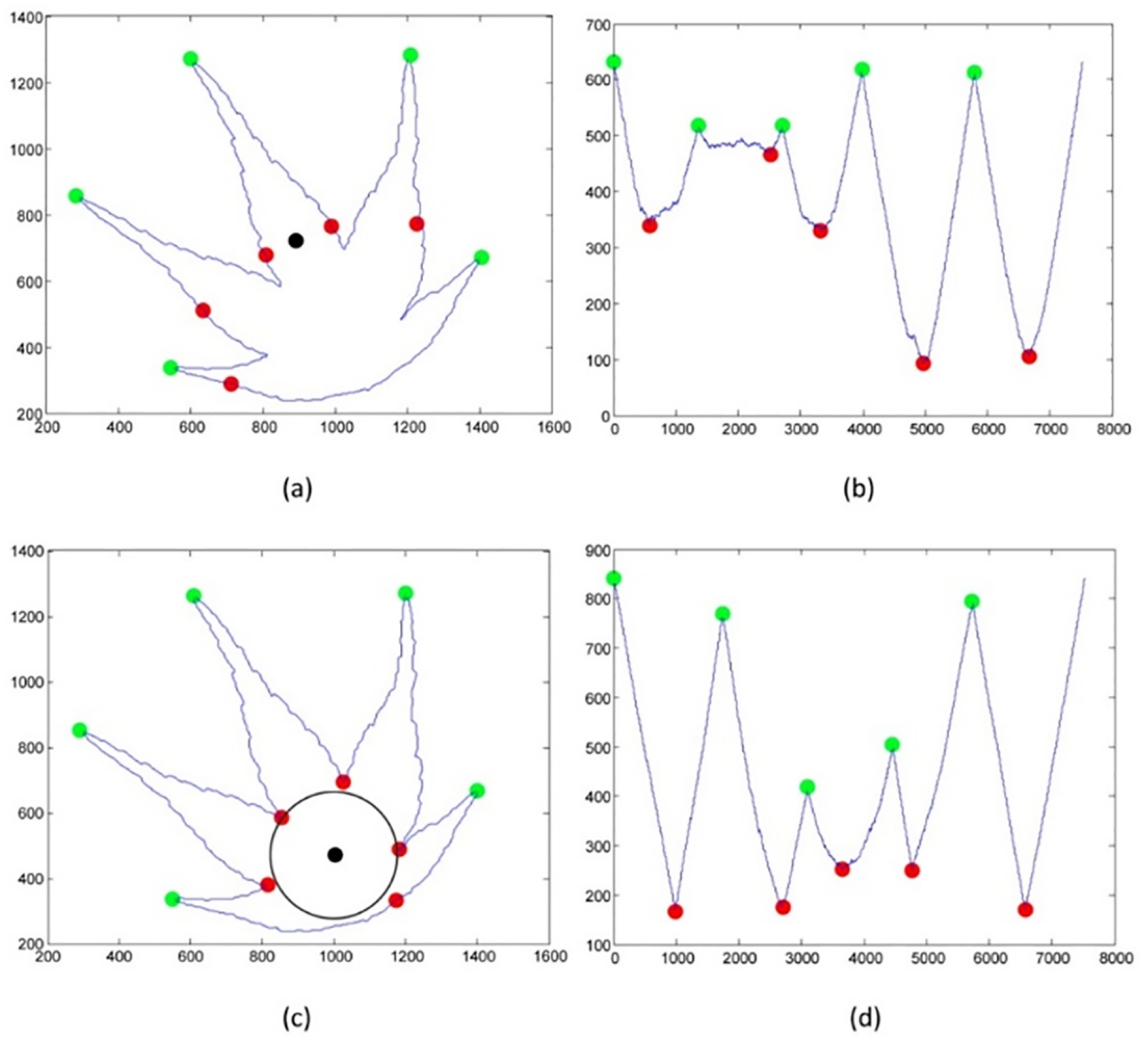
Contour Centroid Distance (a) centroid point get by getting the midpoint of leaf image (b) insignificant shape signature (c) centroid point of incircle in the leaf boundary (d) significant shape signature.

Fig 2 (right) depicts the CCD-EBP in graph. The shape signature is presented, respectably, by using CCD-EBP. In this case, the starting point is not sensitive to the experiment’s outcomes and it can start with any boundary point. CCD-EBP is sensitive to the selection of centroid point. Most of the research undertaken, have appointed the middle point of an object as the object centroid point. However, the correct centroid point should be the centre point of the incircle inside the leaf boundary. The incircle is the largest circle, which fits inside the leaf boundary and just touching the inner edge point in leaf boundary. The miss-located centroid point for Centroid Contour point may lead to spurious peak and valleys as presented in Fig 3(a)–3(d).

Magnitude threshold is used to determine the changes of direction in the CCD-EBP graph. Multiple magnitude thresholds are used to determine the total count of peak or local maxima in the CCD-EBP graph and the valley or local maxima. The total count of peaks and valleys, shown in Fig 3, may vary in magnitude threshold in some of the cases. Therefore, the total count is normalized by getting the most frequent answer from multiple magnitude thresholds.The mathematical term is *Mode* (Eqs 2 to 4). This may avoid the false peaks and valleys, increasing the probability to find the most stale peaks and valleys in CCD-EBP graph.

$${Mthres}_{i}=\{15,25,35,45,55\},\;where\;i\;=\;\{1,2,\ldots ,n(Mthres)\}$$ (2)

$$modPeak\;=\;mode({n(Peak)}_{1},\ldots ,{n(Peak)}_{n(Mthres)})$$ (3)

$$modValley\;=\;mode({n(Valley)}_{1},\ldots ,\;{n(Valley)}_{n(Mthres)})$$ (4)

*Mthres*_*i*_ represents the multiple magnitude threshold. Here, five of the magnitude thresholds are used, which are, 15, 25, 35, 45, and 55. The number of peaks is denoted as *n*(*Peak*)_*i*_ and the number of valleys is denoted as *n*(*Valley*)_*i*_ for every magnitude threshold that is tested. Then the mode of the number of peak and valley for every magnitude is the stable count of peak, *modPeak* and valley, *modValley*. In mathematics, the mode in a set number can be more than one mode. However, in this research, it is limited to one mode, and the first mode is the priority.

**Fig 4:**
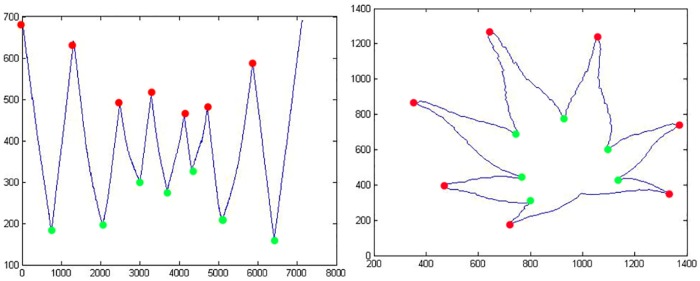
Left: This is the CCD-EBP graph with plotted peak (red) and valley (green), Right: Relocate the peak and valley in the leaf boundary figure.

Fig 4 (left) depicts the peaks and valleys points found in *Acer Palmatum* by using CCD-EBP displayed in graph format. Fig 4 (right) shows the location of peak and valleys of the leaf boundary in image format. The total count of peaks can be directly correlated to the count of the lobes. The valleys represent the location of sinuses.

The local maximum method is a brute force-searching algorithm, which finds the local maximum in a moving window. The window size is determined by a predefined number of local points.

Initially, an *n* point window is placed at the starting point of the data stream. The maximum in this window, as well as its index, is recorded. Then the window is moved one step further. If the new maximum is greater than the saved maximum, it updates both the maximum value and index value and then moves forward a step. If the maximum moves out of the window, i.e., all points in the window are less than the maximum, a peak is found, and the whole window configuration is reconstructed for the next peak. This is summarized in Algorithm 1 and 2.

**Algorithm 1:** Find and relocate the peak and valley point

**Input:** CCD_EBP

**Output:** number of peak, number of valley, position of peak, position of valley

**Begin:**

**Get** Thres[1],…,[n] // here threshold magnitude use is (15, 25, 35, 45, 55)

 **For** index to n

  **Call** FindPeak(Thres[index])

  **Return** number of peak, number of valley, position of peak, position of valley

 **End For**

**modPeak ←** mode(number of peak)

**modValley ←** mode(number of valley)

**indexP ←** find(n(Peak ⩵ modPeak))

**indexV ←** find(nValley ⩵ modValley)

**newPeak ←** Peak(indexP)

**newValley ←** Peak(indexV)

**Plot** Dist versus the sequence number of element in array

**Locate** newPeak and newValley in graph

**Plot** leaf boundary and located the newPeak and newValley

**End**

**Algorithm 2:** Function of finding peak and valley in array

**Function** FindPeak(CCD_EBP)

**Input:** distance of each boundary point with centroid point, Dist

**Output:** Number of peak and valley, location of peak and valley

**Begin:**

**Set** numApex, Peak, Valley **←** 0

**Set** assume the first maximum point is first element in CCD array, mxp **←** *Dist*_1_

**Set** lookformax **←** 1

**Read** Contour Centroid Distance array *Dist*_*i*_

 **For** I = 1 **→** length (Dist) **Do**

  **If** this > mxp

   **Update** mxp **←** this

   **Update** coordinate of mxp

  **End if**

  **If** this <mnp

   **Update** mnp **←** this

   **Update** coordinate of mnp

  **End if**

  **If** lookformax is 1

   **If** this < mxp—delta

    **Compute** Peak ++

    **Set** Lookformax

   **Else**

    **Compute** Valley ++

    **Set** Lookformaxnd **←** 1

   **End if**

  **End if**

** End For**

**Return** total number and coordinate of peak and valley

**End**

#### Apex and base detection

A new framework is proposed to detect the foliage apical extension and basal extension. We identified the maxima curvature for each leaf shape as displayed in Fig 4 (left). The total number of maxima curvatures is the same as the number of lines (*line*_*i*_) and vertex (*V*_*i*_). Each line is connected to the centroid point, *C*(*X*,*Y*) from local maximum points as shown in Eq 5. Each vertex point is formed by two consequent lines, (*line*_*i*_ *and line*_*i*+1_), and *line*_*end*+1_ is the same as *line*_1_. The algorithm then computes the angle of vertex <*V*_*i*_, and angle joint by the above two lines (*line*_*i*_ *and line*_*i*+1_) and sharing a common endpoint located in centroid point (Eqs 6 to 7). The other endpoint of two lines, (*line*_*i*_ *and line*_*i*+1_), are denoted as *k*_*i*_ and *k*_*i*+1_. Both of these endpoints are also the same as the point of the local maxima, which are summarised in Algorithm 3.

$${line}_{i}\;=\;\{\overset{-}{k_{1}C},\;\overset{-}{k_{2}C},\;\ldots ,\;\overset{-}{k_{i}C}\;|i\;=\;1,\ldots ,n\}\;$$ (5)

$$<V_{i}=<k_{i}Ck_{i+1}$$ (6)

$$<V_{i}\;=\;{\text{cos}}^{-1}(\frac{{d(k_{2},c)}^{2}+\;{d(k_{1},c)}^{2}-d{\left(k_{1},\;k_{2}\right)}^{2}}{2\;*\;d(k_{2},c)*d(k_{1},\;c)}),$$ (7)

Where,

$$\text{d(}k_{2},c)=\sqrt{{\left(k_{2}\left(x\right)-c\left(x\right)\right)}^{2}+{\left(k_{2}\left(y\right)-c\left(y\right)\right)}^{2}},$$

$$\text{d(}k_{1},c)=\sqrt{{\left(k_{1}\left(x\right)-c\left(x\right)\right)}^{2}+{\left(k_{1}\left(y\right)-c\left(y\right)\right)}^{2}},$$

$$\text{d(}k_{1},k_{2})=\sqrt{{\left(k_{1}\left(x\right)-k_{2}\left(x\right)\right)}^{2}+{\left(k_{1}\left(y\right)-k_{2}\left(y\right)\right)}^{2}}$$

$$\sum_{i=0}^{i=length(k)}V_{i}=2\pi =360{}^{\circ}$$

**Algorithm 3:** Calculation of each vertex angle

**Input:** local maxima (*k*(*i*)), centroid point (*C*(*X*, *Y*))

**Output:** angle of vertex (<*V*_*i*_)

**Begin:**

 **For** i **←** length (local maxima) +1

  **If** J greater than the length of local maxima

   **J ←** 1 // last line same as first line

  **Else**

   **J ←** i + 1

  **End If**

  **d ←** null

  **d (1) ←** distFunction(*k*_*j*_, *C*)

  **d (2) ←** distFunction(*k*_*i*_, *C*)

  **d (3) ←** distFunction(*k*_*i*_, *k*_*j*_)

  $\text{V}\;(\text{i})\;\leftarrow \;{\text{cos}}^{-1}\left(\frac{\left[{d(1)}^{2}+\;{d(2)}^{2}-\;{d(3)}^{2}\;\right]}{2*\;{d(1)}^{2}*{d(2)}^{2}}\right)$

 **End For**

**End**

**Fig 5:**
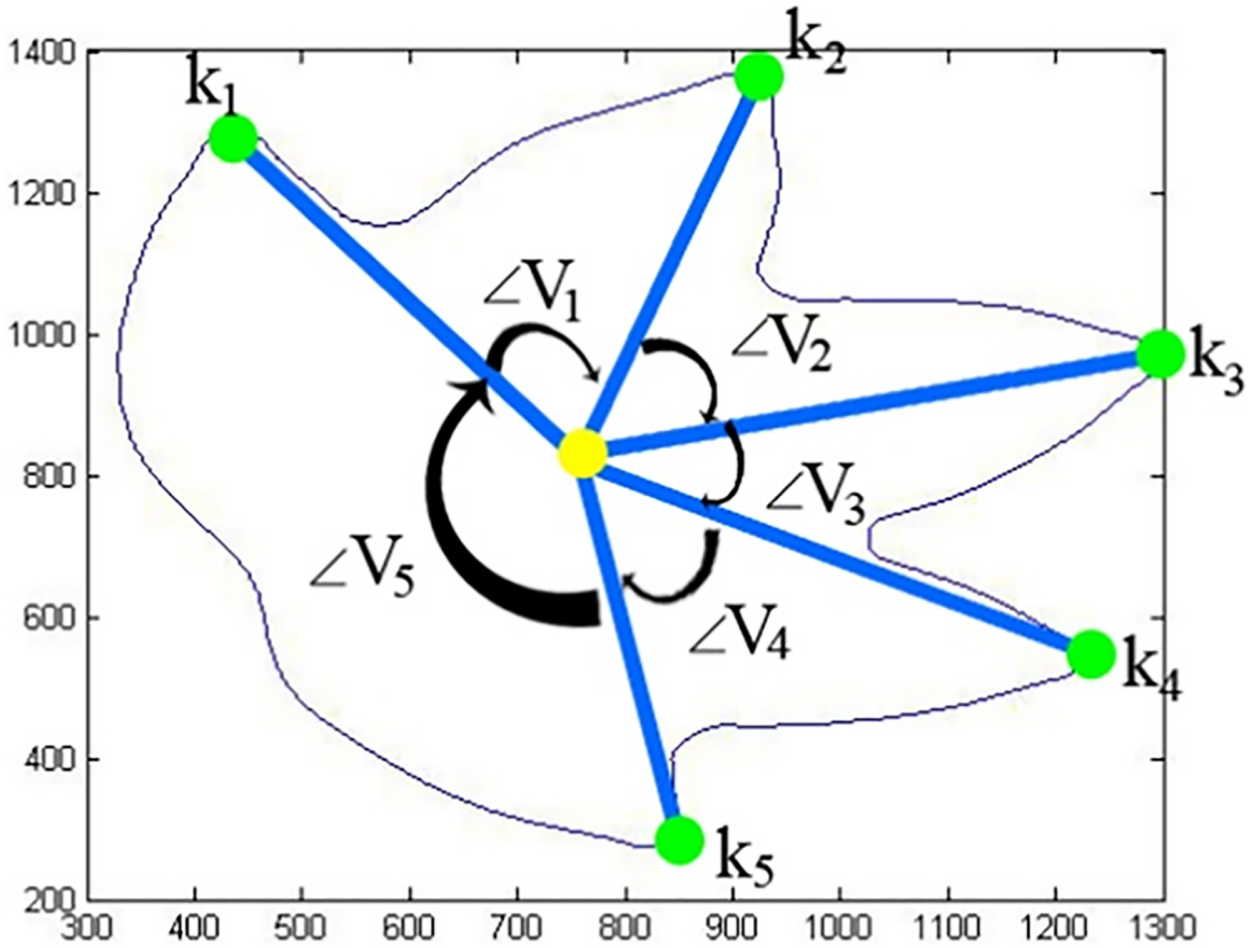
Label of local maxima and angle of vertex.

After identifying the lines (*line*_*i*_), local maxima (*k*_*i*_), and the angle of vertex (<*V*_*i*_), the local maxima is categorized into two major regions, which are denoted as north region, and south region. The angle of vertex (<*V*_*i*_) is used to group them either to north region or south region (since the apex and the base are in opposite sites, so divided into south and north can detect the leaf apex and base easily). Among the local maxima, one of them is the terminal leaf apex and the opposite valley or peak is known as ‘*leaf base*’. Leaves are symmetrical, therefore, the angle of the vertex found, can be used to differentiate the south region and north region as shown in Fig 5.

$$V_{norm}\left(j\right)=\frac{V_{j}-\text{min}\left(V\right)}{\text{max}\left(V\right)-\text{min}\left(V\right)},j=\left\{1,\ldots ,length\left(i\right)\right\}$$ (8)

$$V_{region}\left(j\right)=\{\begin{array}{c} V_{norm}\left(j\right)>Thres_{norm},string\left(north\right)\\ \begin{array}{cc} otherwise & ,string\left(south\right) \end{array} \end{array}$$ (9)

Parameter *V*_*norm*_ represents the normalization of the angle of vertex (∠*V*_*i*_) and parameter j is index of the current ∠*V* as shown in Eq 8. Parameter *V*_*j*_ represents the current ∠*V*. Where min(V) represents the minimum angle among the ∠*V* in a leaf image. Parameter max(V) represents the maximum angle among the ∠*V* in a leaf image. Parameter *V*_*region*_(*j*) are the group that particular angles of vertex (∠*V*_*i*_) to either “north” or “south” as shown in Eq 9. Parameter *Thres*_*nom*_ is a predefined value. However, in this research, 0.5 is used as the predefined value.

**Fig 6:**
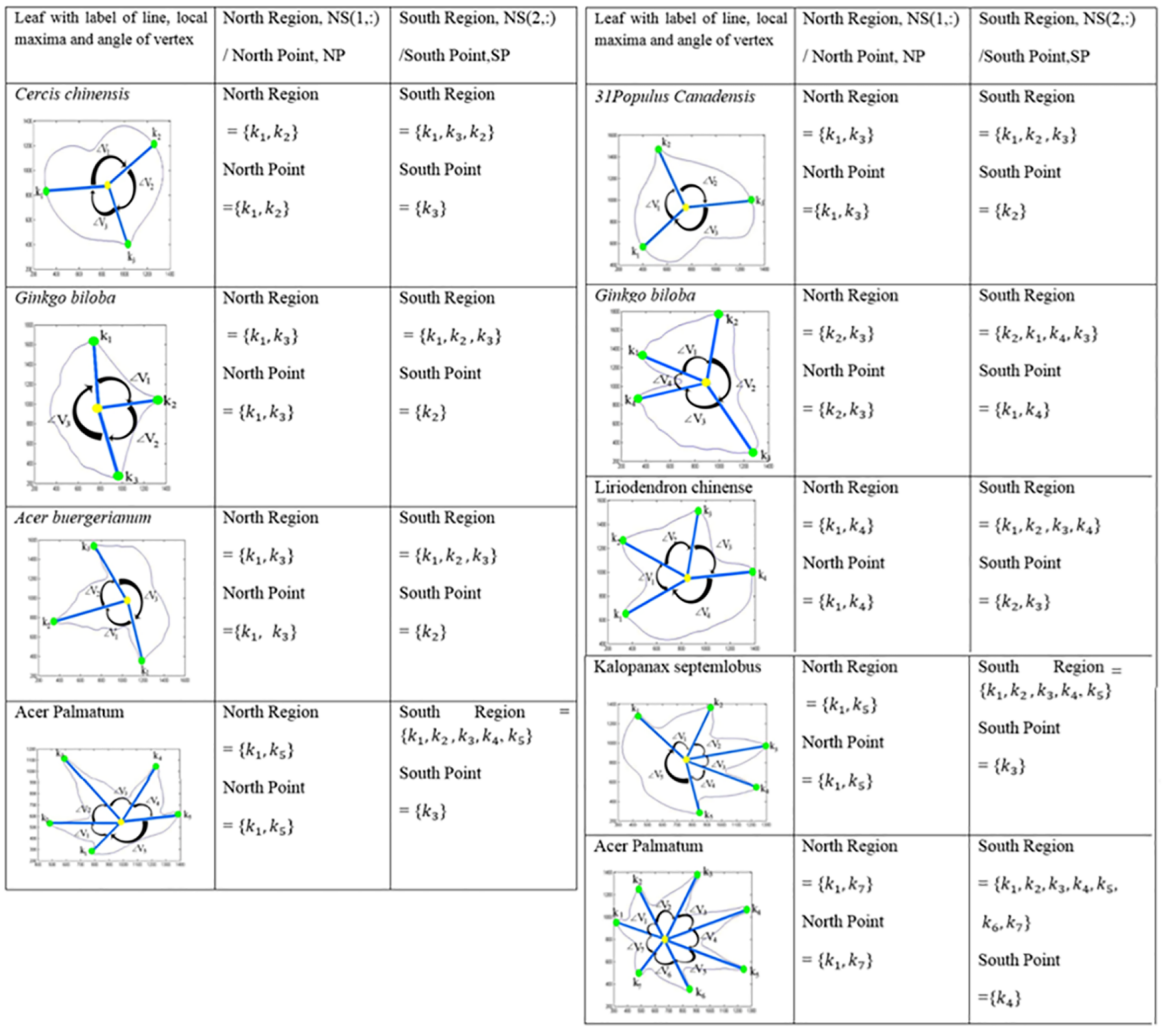
Location of local maxima into north region or south region.

Parameter *NS*(1,:) stores the local maxima points which ∠*V*_*i*_ is belonging to north region and *NS*(2,:) belonging to south region. However, since the vertex point is forming by 2 and contains local maxima (*k*_*i*_) and (*k*_*i*+1_), therefore, these two continuing local maxima points are grouping in the same angle of vertex (∠*V*_*i*_). For example, in Fig 6, the local maxima in NS(1,:) are *k*_1_ and *k*_5_, the local maxima in NS(2,:) are *k*_1_, *k*_2_, *k*_3_, *k*_4_ and *k*_5_.

After dividing the local maxima (*k*_*i*_) into 2 groups, north and south region, next determine the north point and south point. Since the leaf is symmetric, therefore, the median of local maxima will be north point (*NP*_*i*_) or south point (*SP*_*i*_), however, for those regions which have even number of local maxima, meaning, two of the local maxima will be the north point (*NP*_*i*_) or south point (*SP*_*i*_). These finding points are presented in Algorithm 4.

**Algorithm 4:** Finding south point and north point

**Input:** Angle of vertex (V)

**Output:** North point (NP), South point (SP)

**Begin:**

 **For** j **←** length of vertex

  //normalize angle of vertex

  V_norm(j) **←** (*V*_*j*_ − min(V)) / (max(V) − min(V))

  **IF** (*V*_*norm*_ (j) >*Thres*_*norm*_)

   *V*_*region*_ (j) **←** “north”

  **Else**

   *V*_*region*_ (j) **←** “south”

  **End IF**

 **End For**

 **For** i **←** length of vertex

  **IF** (i equal to length of vertex angle)

   J **←** 1

  **Else**

   j **←** I +1

  **End IF**

  **IF** (*V*_*region*_(i) equal to “north”)

  //NS(1,:) refers to the north array

   NS(1,i) and NS(1,j) **←** 1

  **Else**

   NS(2,i) and NS(2,j) **←** 1

  **End IF**

  **If** (n(NS(2,:) ⩵ 1)> n(NS(1,:) ⩵ 1))

   *NS*_*new*_(2, :) **←** NS(2, :) –NS(1, :)

   *NS*_*new*_(1, :) **←** NS(1, :);

  **Else**

   *NS*_*new*_(1, :) **←** NS(1, :) –NS(2, :)

   *NS*_*new*_(2, :) **←** NS(2, :);

  **End IF**

  **Find** index which *NS*_*new*_(1, :) equal to 1

  North **←** K(index);

  **Find** index which *NS*_*new*_(2, :) equal to 1

  South **←** K(index);

 **End For**

 Median **←** null

 **If** (n(North) % 2 ⩵ 0) //even number

  Median(1) **←** n(North)/2

  Median(2) **←** n(North)/2 + 1

 **Else** //odd number

  Median(1) **←** ceil(n(North)/2)

 **End IF**

 NP **←** North(Median)

 **If** (n(South) % 2 ⩵ 0) //even number

  Median(1) **←** n(South)/2

  Median(2) **←** n(South)/2 + 1

 **Else** //odd number

  Median(1) **←** ceil(n(South)/2)

 **End IF**

 SP **←** South(Median)

**End**

**Fig 7:**
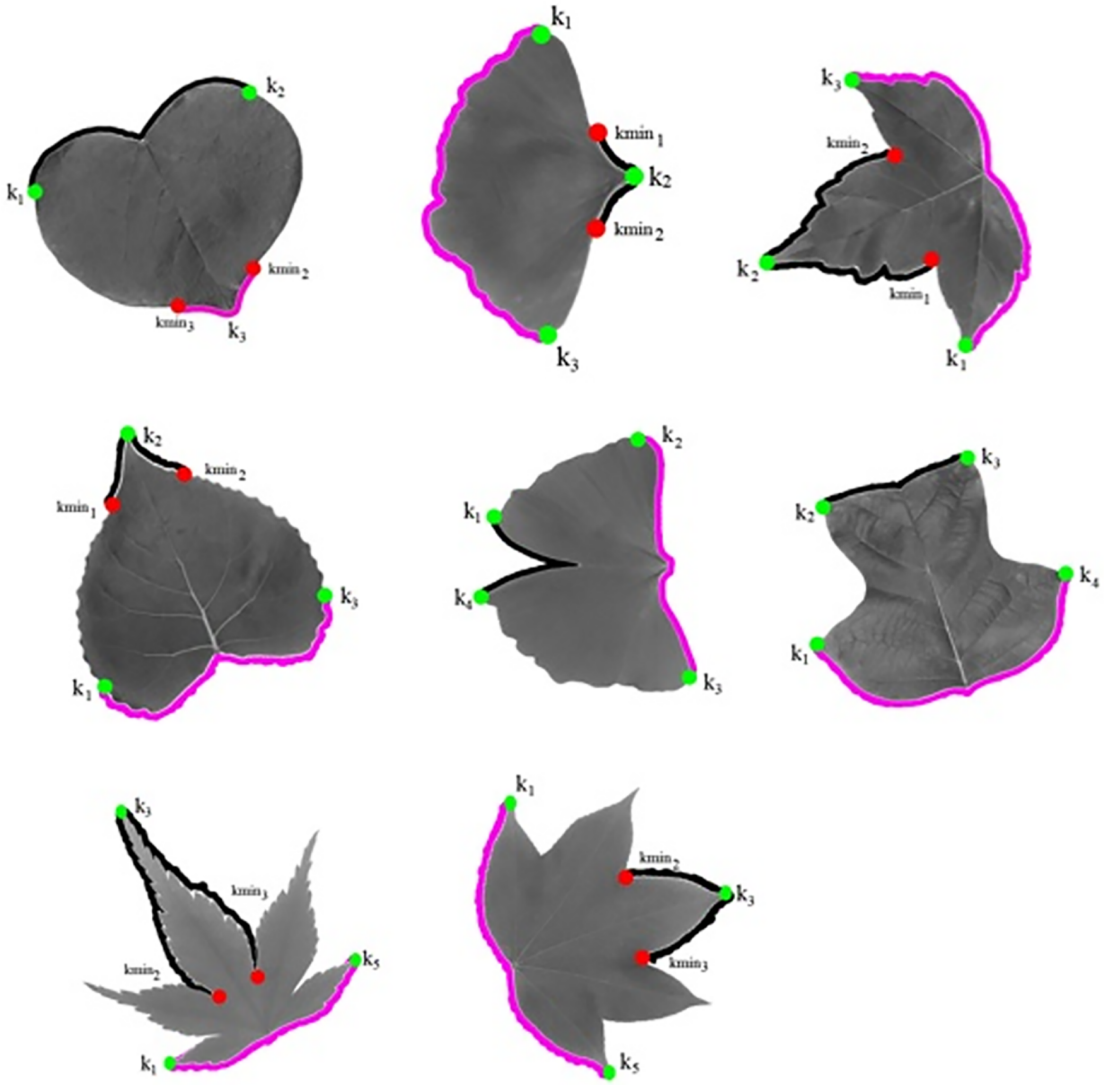
South part and north part (partNP and partSP).

For the north point (*NP*_*i*_) or south point (*SP*_*i*_), which only had a single point, the insertion of local minima is needed. Two local minima are located separately in the left and right side of the local maxima. Fig 7 shows the insertion of local minima into local maxima. *UniqueCur*(*noCur*) is the leaf contour in clockwise direction. Therefore, it eases the way to extract the apex curve and base curve. Since it is not known whether the south or north region is the apex, so it is temporary denoted as north part (*partNP*) and south part (*partSP*). Eqs 10 and 11 show the extraction of north part and south part.

$$partNP\{:\}\;=\;UniqueCur({index(Kmin}_{4}):\;index({Kmin}_{5}))$$ (10)

$$partSP\{:\}\;=\;UniqueCur(index(k_{1}):index(k_{2}))$$ (11)

However, the detected parts (*partNP* and *partSP*) encounter confusion about whether which part is the leaf apex and which part is the leaf base. For the leaf sample, which comes together with the leaf petiole, it can be easily differentiated. However, for the dataset without the leaf petiole it had difficulty in identifying the leaf apex and the leaf base.

**Fig 8:**
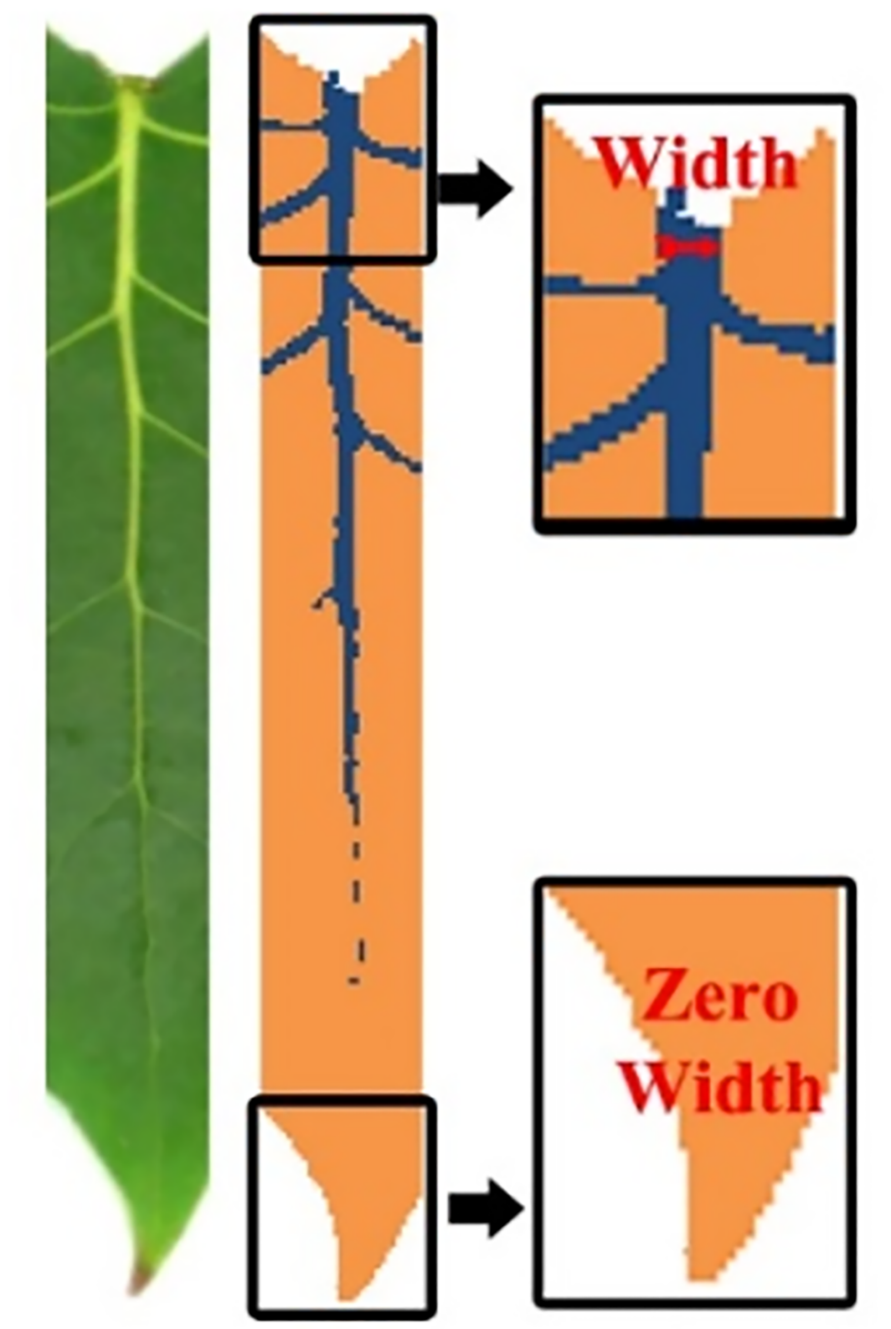
Example of mid-vein’s diameter near to leaf apex and base.

To simplify the cascading process of analysis, a rotation is applied to the sample leaf, based on the detected north and south point. After the rotation, the width of mid-vein is computed. The width of the mid-vein is used to differentiate whether the north part or south part is the foliage base or the foliage apex. The size of mid-vein, which detached to the petiole is wider compared to the mid-vein in the foliage apical (Fig 8). The foliage apex and base is presented in Algorithm 5.

**Algorithm 5:** Find the foliage apex and base

**Input:** Foliage sample, south point, north point, local maxima, local minima

**Output:** Foliage apex and base, Align sample

**Begin:**

RotImage **← Rotate** Foliage sample (north point is pointed upward)

CropVein **← Crop** Foliage sample which had mid-vein

**Resize** CropVein

**Compute** the hue of the CropVein

**Discrete** Hue and find the colour coherent vector based on the intensity different

**Compute** the width of foliage mid-vein

 **IF** width of mid-vein in south point is widen than mid-vein in north point

  South point **←** foliage base

  North point **←** foliage apex

  Align sample **←** RotImage

 **Else**

  North point **←** foliage base

  South point **←** foliage apex

  Align sample **←** rotate(RotImage, 180)

 **End IF**

**End**

#### Leaf margin detection

The leaf margin is also known as the leaf edge or leaf blade. The leaf margin is another important part, which possesses a unique feature to represent plant species. The method used to detect the leaf margin is similar to the previous methodology. The only difference between these two methodologies are the magnitude threshold value applied in CCD-EBP.

The magnitude threshold in this section had applied with lower magnitude threshold of 2’s, and used to detect slight curvature changes. However, the lower magnitude threshold detects the leaf apex beside leaf teeth. Therefore, the detected small local maxima and small local minima had to exclude the leaf apex (Eqs 12 to 13).

LeafTeethPeak=smalllocalmaxima-localmaxima (12)

LeafTeethValley=smalllocalminima-localminima (13)

Where,

smalllocalmaxima=Peak(>SmallThreshold)

smalllocalminima=Valley(<SmallThreshold)

**Fig 9:**
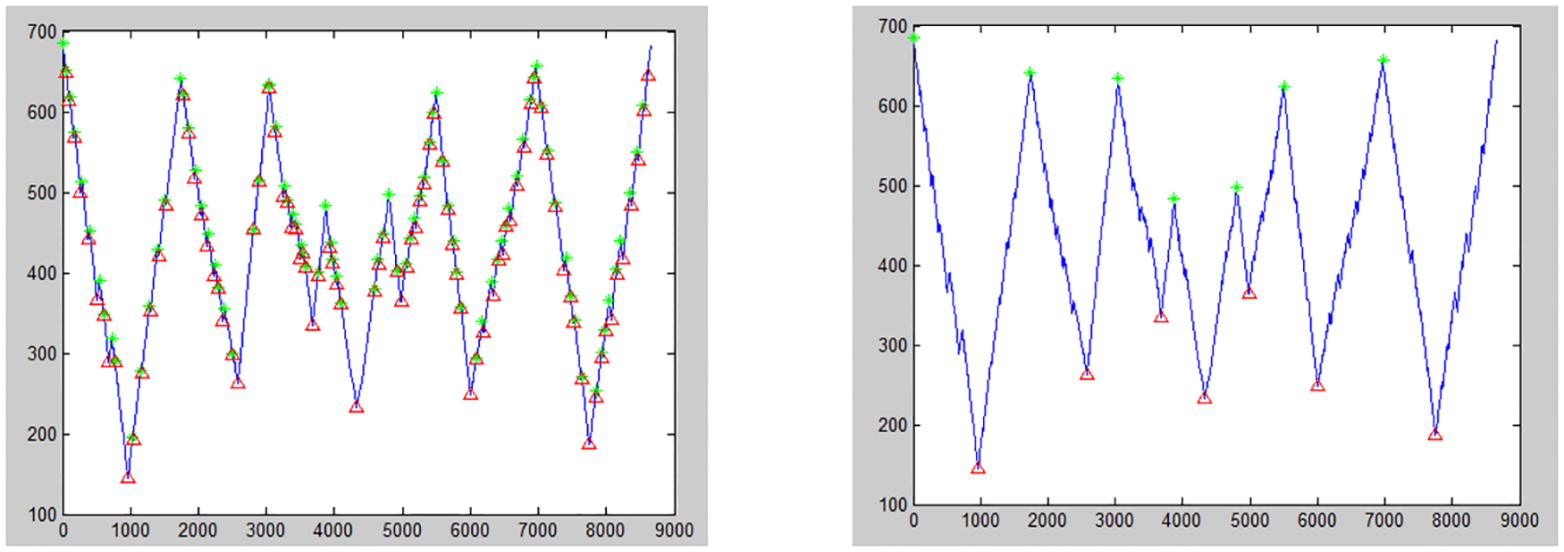
Graphs of Contour Centroid Distance for Every Boundary Point (a) CCD-EBP in graph with small local maxima and small local minima (b) CCD-EBP in graph with local maxima and local minima.

**Fig 10:**
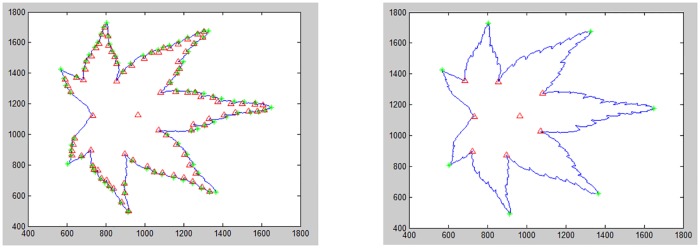
Left: Relocate the small peak and small (small magnitude threshold) valley in leaf boundary figure, Right: Relocate the peak and valley (large magnitude threshold) in leaf boundary figure.

The smaller the magnitude, the smaller the peaks and valleys are classified. However, this statement is not suitable for 1’s as magnitude threshold, as 1’s is too small and easily detects unwanted zig-zags as fault leaf teeth. Thus the best-fit small magnitude threshold of 2’s, which covers most of the leaf teeth but avoids unwanted zig-zags, is used. See Algorithm 6. Fig 9 exhibits the comparison of magnitude threshold used in CCD-EBP graph and Fig 10 relocates the founded peaks and valleys in leaf boundary image.

**Algorithm 6:** Leaf margin detection

**Input:** CCD_EBP

**Output:** position of teeth peak, position of teeth valley

**Begin:**

**Get** SmallThres[1],…,[n]

 **For** index to n

  **Call** FindPeak(SmallThres[index])

  TeethPeak **←** small peak—peak

  TeethValley **←** small valley—valley

 **End For**

**End**

**Algorithm 7:** Leaf margin detection

**Input:** CCD_EBP

**Output:** position of teeth peak, position of teeth valley

**Begin:**

**Get** SmallThres[1],…,[n]

 **For** index to n

  **Call** FindPeak(SmallThres[index])

  TeethPeak **←** small peak—peak

  TeethValley **←** small valley—valley

 **End For**

**End**

### External leaf structure feature extraction

Feature extraction from apex, based and margin is taken into account in this section.

#### Leaf apex and leaf base feature

**Fig 11:**
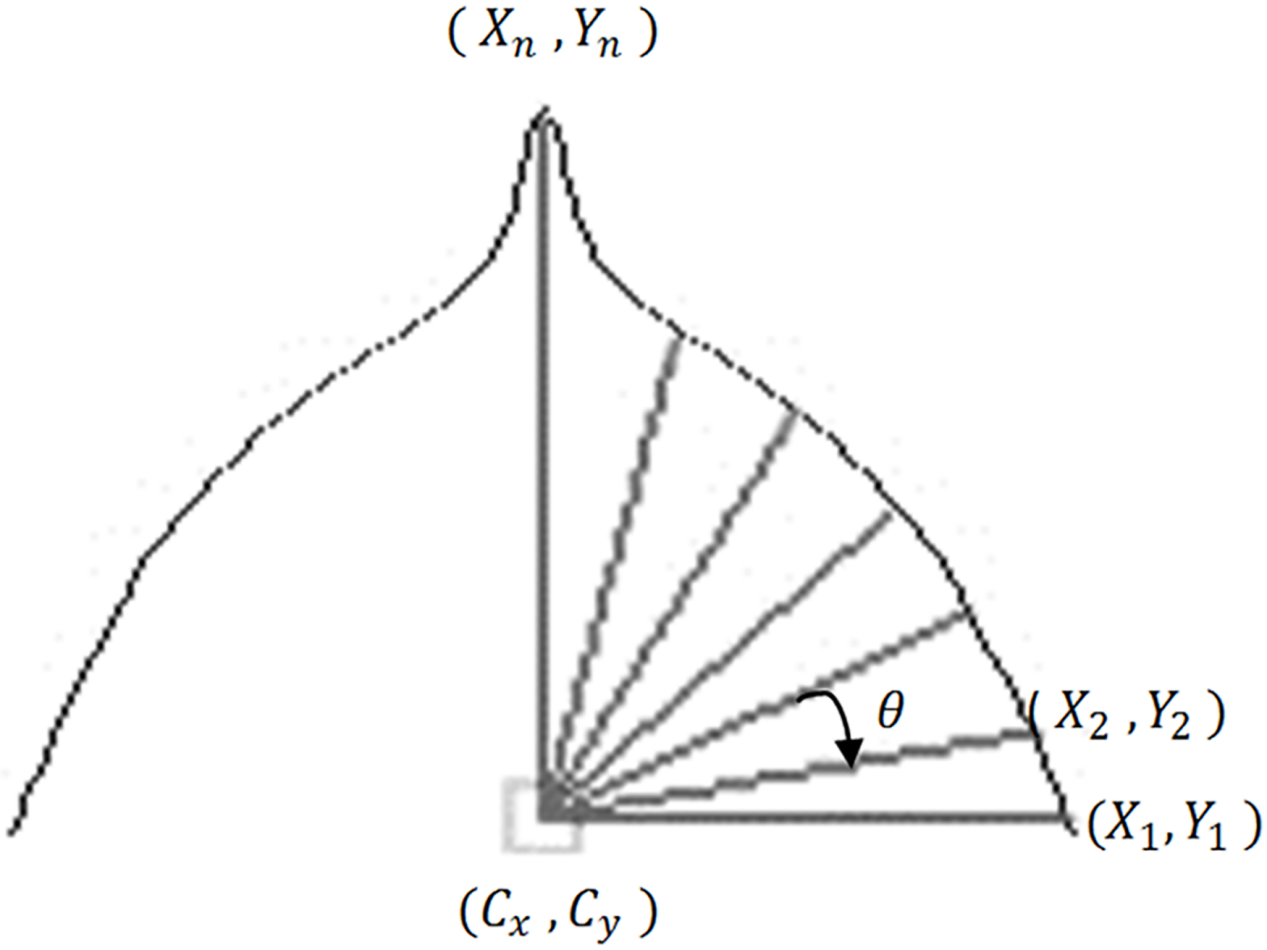
Centroid Contour Gradient approach.

Centroid Contour Gradient (CCG) is used to compute the gradient value of a continuing leaf apex boundary point corresponding to the interval angle, θ (Fig 11). This method has the ability to obtain the curvature information of the leaf. This method is suitable to capture the description of the leaf tip and the leaf base. The leaf tip is usually defined as the top of the leaf and the leaf base. In fact, the leaf tip can be divided into acuminate, acute, cuspidate, obtuse, and truncate. The leaf base can be divided into acute, cuneate, rounded, and oblique. Using this approach, the type of the leaf tip and the leaf base can be discerned.

Although there are a series of leaf apex and base boundary points, but only the boundary points corresponding to its interval angle, *θ*, is chosen. The selected boundary points are noted as (*X*_*i*_ and *Y*_*i*_) and (*i* = 1, 2, …, n-1, n). Here, *n* represents the number of intervals that is given by *n* = (90 + *θ*)+1. Only the shape description for right side of leaf tip is captured as the leaf part of leaves is actually the symmetrical to its right part. Hence, their gradient should be the same, so it is not necessary to do redundant work.

For example, if 15 degrees is selected as our default angle, this means that only selecting the pixels on the leaf boundary point at different angle set *θ* = {0, 15, 30, 45, 60, 75, 90} is enough. The only leaf boundary point is selected if they fit in Eq 14.

$$Y_{i}\;=\;[\text{tan}\left(\theta \right)*(X(i)-\;C_{x})]+\;C_{y}$$ (14)

Co-ordinate (*C*_*x*_, *C*_*y*_) represents the centroid point of the leaf tip. After obtaining the boundary points which intersect with the respective angle, calculate the positive gradient between the continued 2 boundary points in the corresponding angle, i.e. (*X*_2_, *Y*_2_) and (*X*_1_, *Y*_1_), (*X*_3_, *Y*_3_) and (*X*_2_, *Y*_2_)… (*X*_*i*+1_, *Y*_*i*+1_) and (*X*_*i*_, *Y*_*i*_) using (Eq 15).

$$G_{i}\;=\;|\frac{Y_{i+1}-\;Y_{i}}{X_{i+1}-\;X_{i}}|,\;i\;=\;\{1,2,3,\ldots ,n-1\}$$ (15)

This method is derived from the existing widely used framework; Centroid Contour Distance (CCD) approach. The difference between these two approaches are; Centroid Contour Distance (CCD) is used to compute the distance from centroid point to the pixels on the leaf’s contour which corresponds to the threshold angle set, and for Centroid Contour Gradient (CCG), it is used to calculate the positive gradient value between two of the consequent leaf’s contour points, corresponding to the interval angle set. In this research, the novel method (CCG) is used to describe the information of the leaf tip and the leaf base.

#### Leaf margin feature

The leaf margin refers to the leaf blade, side, or edge of the leaf. The leaf margin can be described by using morphology of the leaf teeth. In this research, we used ripples pixel area, CCD-EBP and curvature maxima and curvature minima to capture the characteristic of the leaf margin. The margin with trichomes (plant hairs) are too small to detect, therefore, they are excluded in this research and detected as complete. For example, the plant species *phyllostachys edulis (Carr*.*) Houz* have margin ciliate, however, the trichomes are unseen so in this research, they are classified as complete.

**Fig 12:**
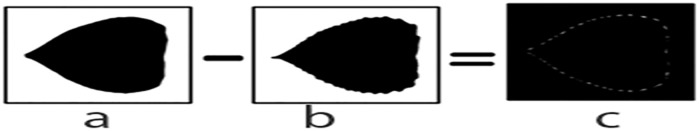
a)Binary image of smoothing edge by using disk filter b) Binary image of original leaf sample c) Ripples are 
$$RipplesRatio\;=\;\frac{RipplesArea}{LeafArea}.$$(16)

The first step, the ripples pixel area of leaf margin are found by finding the difference of binary image from smoothing leaf edge using a filter and then binarizing the original leaf samples (Fig 12). The total white pixel count in the ripples area image is computed. Then find the ratio of ripples area over the total black pixel in binary image is calculated (Eq 16).

**Fig 13:**
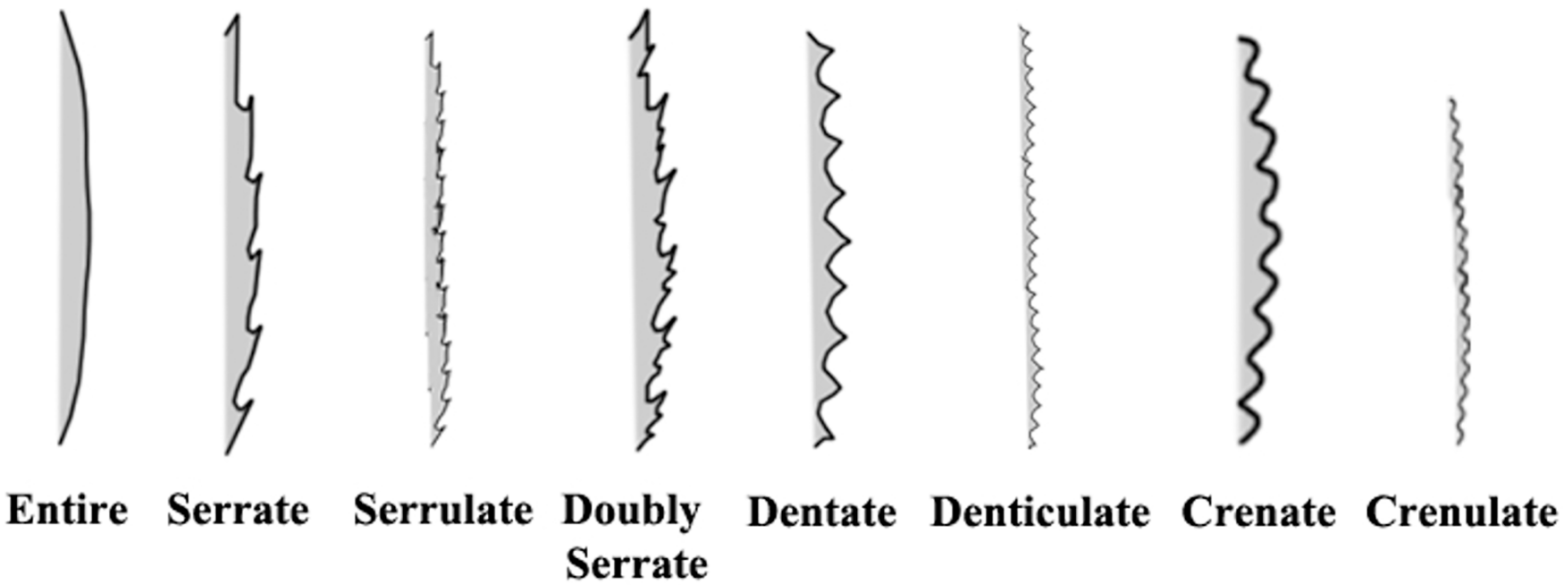
Leaf margin type.

The RipplesRatio approximates to zero, meaning that the leaf margin is complete, otherwise, the leaf margin possesses leaf teeth. Leaf teeth can be divided into 8 groups,serrate, serrulate, doubly serrulate, dentate, denticulate, crenate, and crenulated. The morphology of serrate and serrulate are actually the same, their teeth are a saw-like shape. The only difference is the margin with serrulate which is the diminutive of serrate or it can be called small serrate. Denticulate is also the diminutive of dentate. The shape of the denticulate and dentate look like shark teeth. The shape has approximately equal length at both sides of teeth. Crenate and crenulated have approximately equal length for both sides. These two types of leaf teeth are rounded. Crenulated is the diminutive of crenate. According to Simpson (2011), serrate, crenate, dentate is $\frac{1}{16}$ to $\frac{1}{8}$ of distance to the midrib, however, serrulate, denticulate, crenulated is cutting to $\frac{1}{16}$ from the midrib distance (Fig 13).

The ratio of diminutive teeth (*RatioDT*) can be obtained by getting the ratio of teeth’s length to the length of the teeth to the midrib (*LTeeth2Midrib*). If the ratio of diminutive is less than the ratio one sixteenth ($\frac{1}{16}$), these teeth are considered as diminutive teeth or also called small teeth. If *LTeeth2Midrib* are greater than $\frac{1}{16}$, the leaf teeth are considered as big teeth. Eqs 17 to 18 explains the statement above,

RatioDT=Lteeth/LTeeth2Midrib (17)

$$diminutiveTeeth\left(x\right)=\{\begin{array}{c} true,\;x<\frac{1}{16}\\ false,x>\frac{1}{8},\;x\leq \frac{1}{16} \end{array}$$ (18)

**Fig 14:**
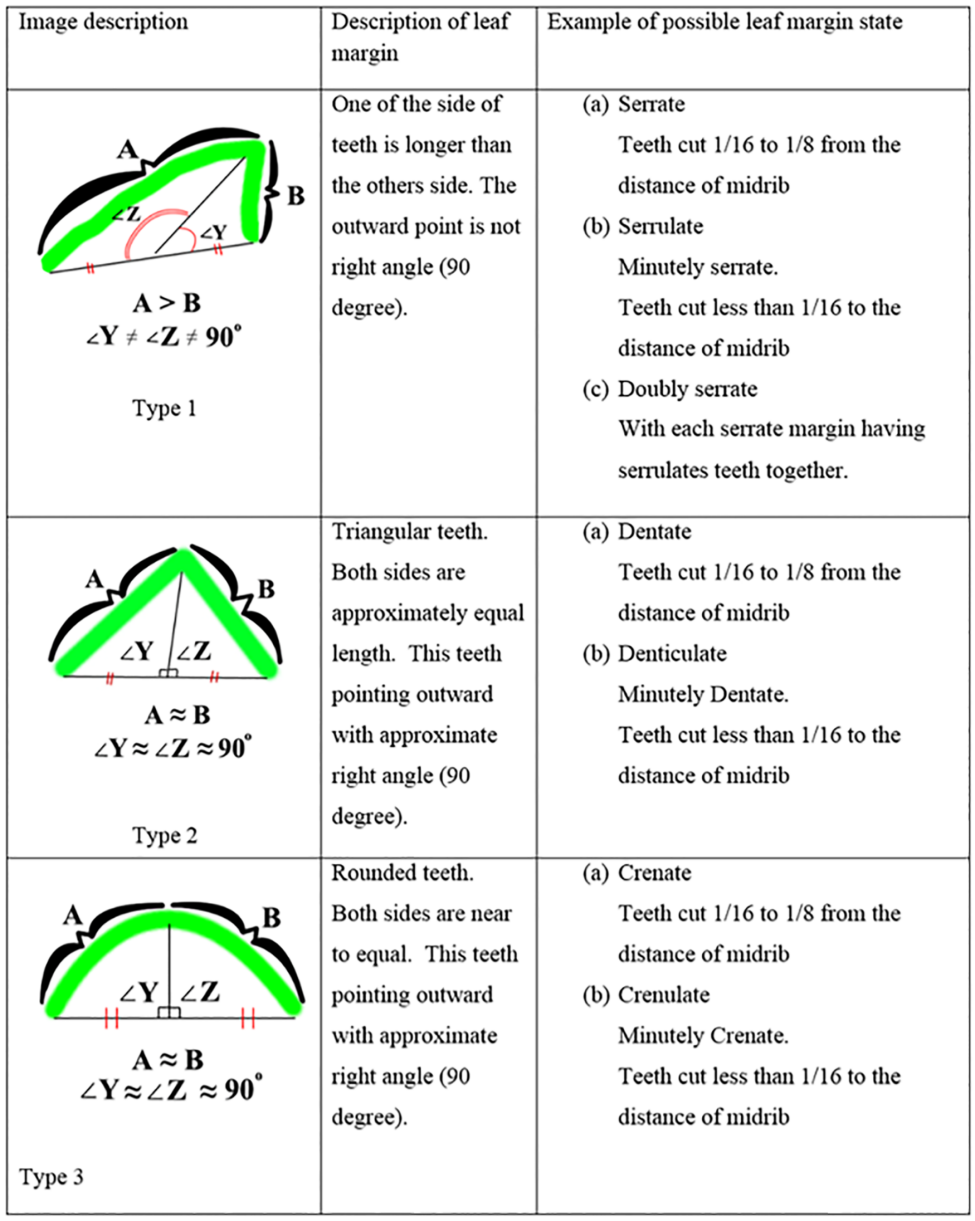
Description of leaf margin type.

The curve represents (in Fig 14) the single leaf teeth. Single teeth are divided into 2 curves starting from outward point and end in a dented point of leaf tooth. Both points are denoted as ‘A’ and ‘B’. The outward point is the curvature maxima of the curve and the dented point of leaf tooth is the curvature minima of the curve. The length of ‘A’ and ‘B’ are used to differentiate the leaf teeth type. If ‘A’ and ‘B’ have approximate equal length, which means the possible leaf type is Type 2 (dentate, denticulate) and Type 3 (crenate and crenulate). Otherwise, the possible leaf type is serrate, serrulate, and double serrate (Type 1). Eq 19 explains the above statement.

$$TeethType=\{\begin{array}{ll} Type\;2,3, & A/B\approx 1\\ Type\;1, & Otherwise \end{array}$$ (19)

By using the length of ‘A’ and ‘B’, teeth type of 2 and 3 are separable. The teeth of type 2 are triangular in shape and the teeth of type 3 are rounded. Triangularity is used to differentiate them (Eqs 20 and 21). If the area of single teeth is greater than the area of triangle, which means that the teeth is rounded, as the rounded teeth have larger area compare to triangular.

$$triangularity\;=\;\frac{Area\;of\;single\;Teeth}{Area\;triangular}$$ (20)

$$f\left(x\right)=\{\begin{array}{l} Type\;2,\;Triangularity\leq 1\\ Type\;3,\;otherwise \end{array}$$ (21)

If the leaf teeth is type 2, the possible leaf margin state are dentate and denticulate. If the Boolean value of diminutive teeth is ‘true’, the leaf margin teeth is denticulate, otherwise it is dentate. In the same way, the leaf teeth with type 3 applied the same method. If the diminutive teeth is ‘true’ for leaf teeth type 3, the possible leaf margin state is crenulated, or else the possible leaf margin state is crenate. Fig 14 outlines the description of leaf margin type.

## Results and discussion

### Plant identification based on external leaf structure

This section carries out the detection of external leaf structure, and includes the detection of local maxima, local minima, leaf boundary, apex, base, margin, and venation. The characteristic of every part of the leaf in each plant species is stated in a botanical terminoligy. The characteristic of each plant species are described based on the information of the well-established Electronic Data information Source (EDIS) that operates since 2003.

### Leaf apex and base detection and characteristic state

**Fig 15:**
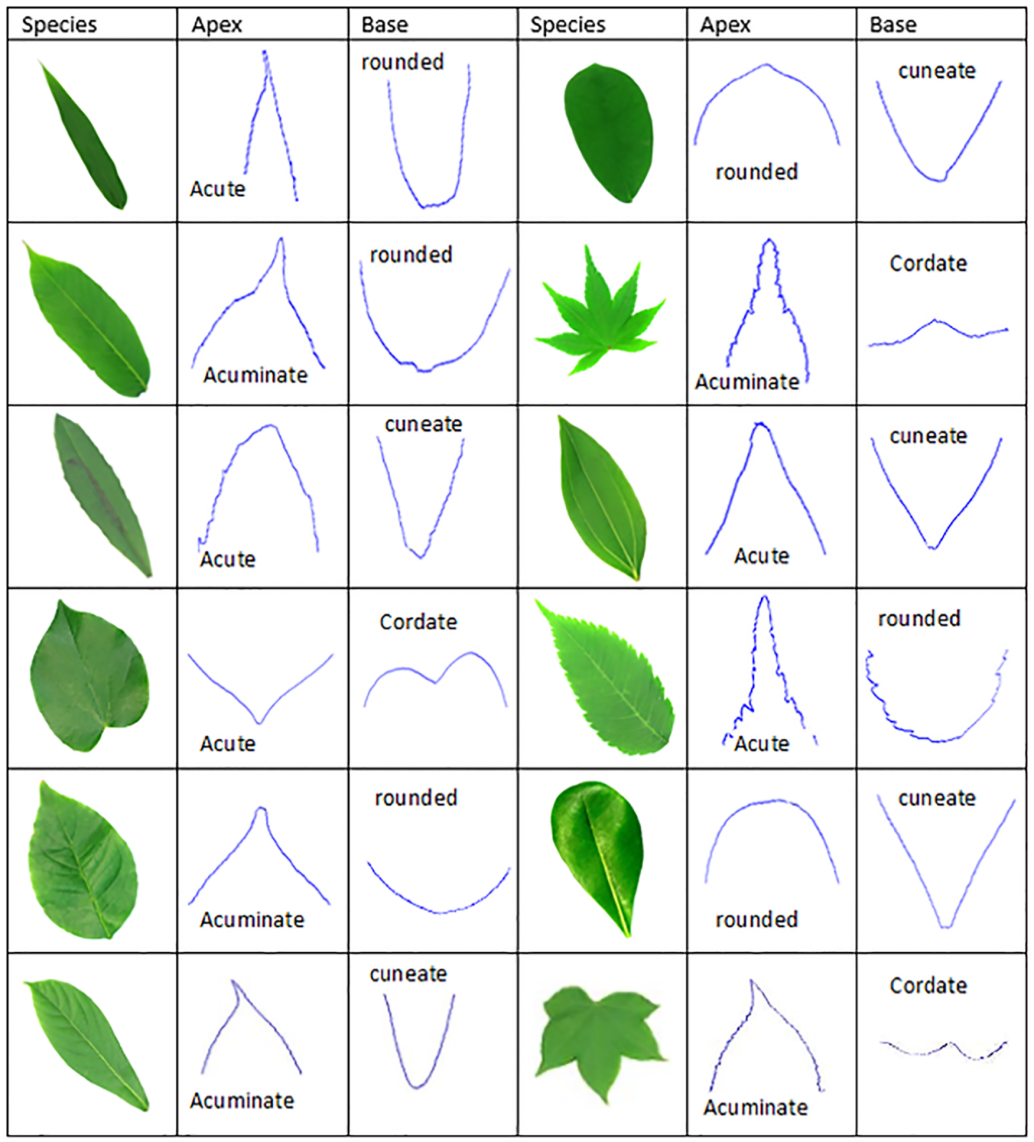
Apex and base detection and their botanical characteristic in Flavia dataset.

**Fig 16:**
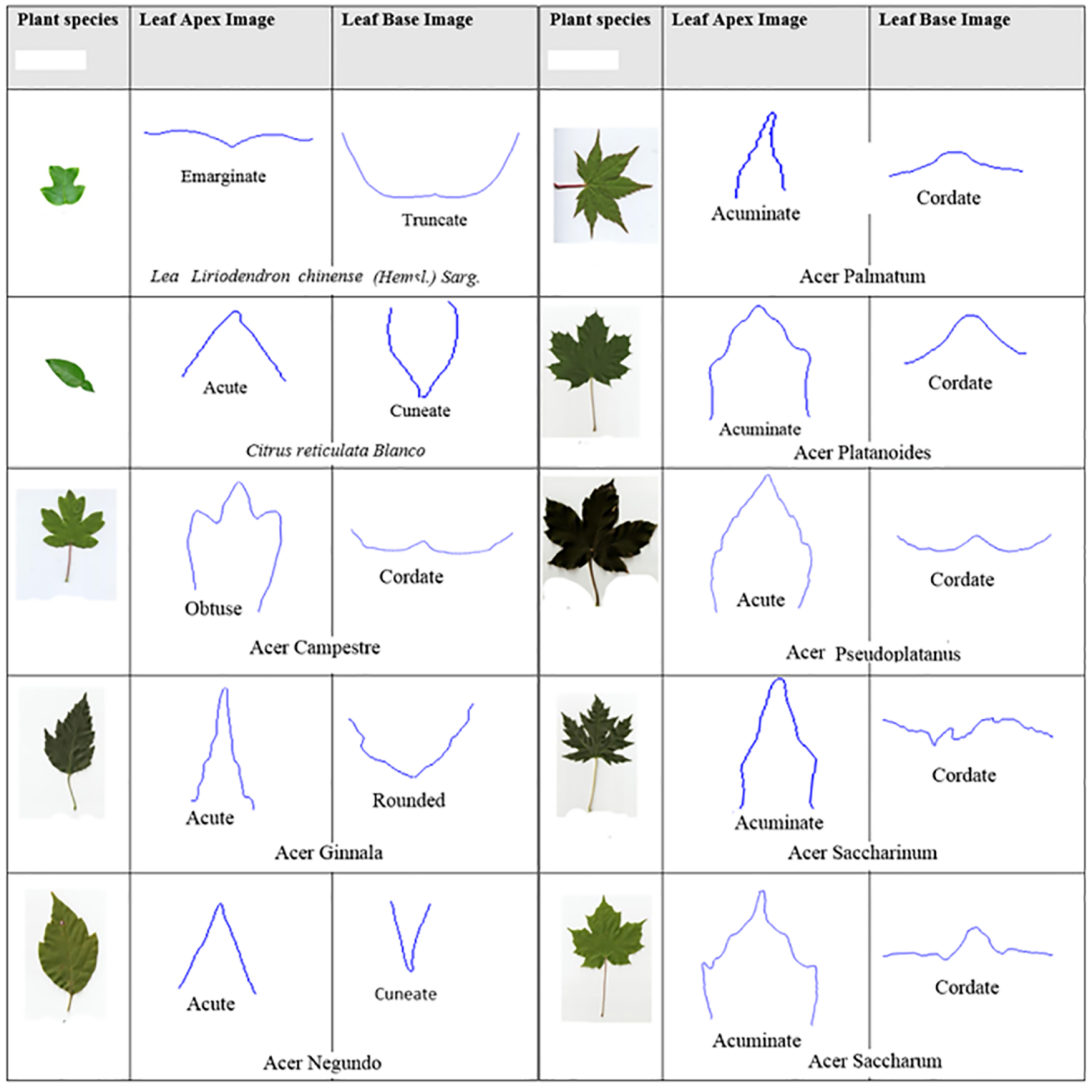
Apex and base detection and their botanical characteristic in Acer dataset.

From the results found in local maxima and local minima, leaf apex and base are then determined. The curvature of the leaf apex and base for each plant species in the Flavia dataset and the Acer dataset are showcased in Figs 15 and 16 respectively.

If the margin of the apex is abruptly incurved and its angle is less than 45 degrees, it is called Acuminate apex. For the apex which had an almost straight side with the intersection angle between 45 degree and 90 degree, is classed as an acute apex. The margin of the rounded apex are curved to form a smooth arc.

The leaf base with its margin are has an angle between 45 degrees to 90 degrees. The margin of the rounded base are approximately curved to form a single smooth arc. The cordate base are valentine-shaped with two rounded margins.

### Leaf margin detection and characteristic

**Fig 17:**
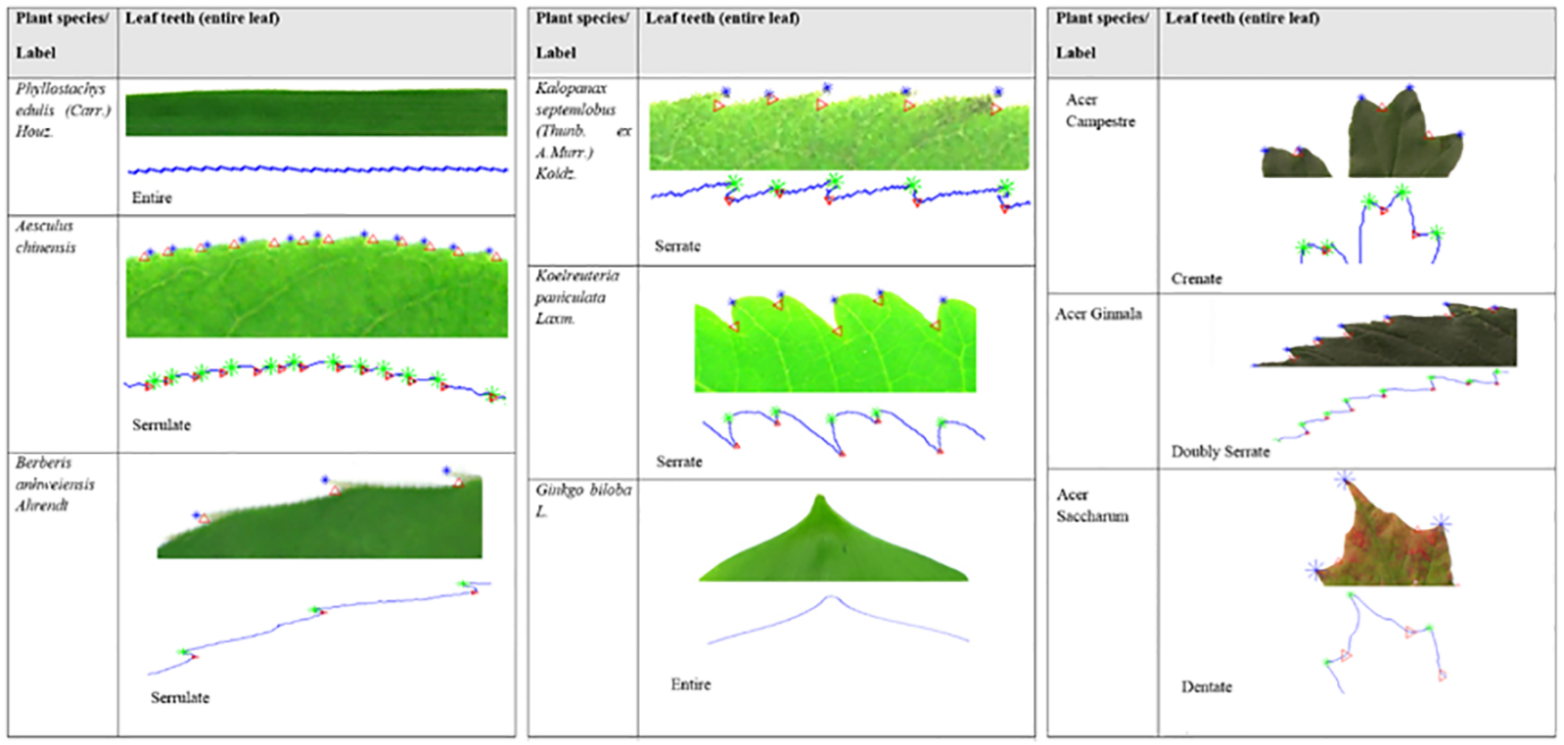
Teeth features for each plant species in Flavia and Acer dataset.

Teeth features for each plant species are discussed in this section. The outward teeth are labelled with a star point (*) and the inward teeth are labelled with a triangle (Δ). The ground truth and predicted classification of some of the leaf margin in Flavia and Acer datasets are presented in Fig 17.

### The results of previous work

The proposed method outperformed the previous works in term of the number of dataset used and the accuracy as more external leaf structure features are applied. Table 1 shows that the more features that are integrated in leaf identification, the higher the accuracy are. However, the selection of features plays a key role. There is 94.76%accuracy achieved using the proposed method. The accuracy equation is as shown as below (Eq 22).

$$Accuracy=\frac{\text{total}\;\text{number}\;\text{of}\;\text{query}}{\text{relevant}\;\text{number}\;\text{of}\;\text{images}}$$ (22)

**Table 1:** Comparison of previous proposed method with our proposed method for Flavia dataset.

Author	Features	Accuracy	Dataset	Training	Testing	Species
**Satti, Satya and Sharma (2013)**	Shape, colour	93.3%	1907	1742	165	33
**Chaki, Parekh and Bhattacharya (2015)**	Shape, texture	87.1%	930	620	310	31
**Arun, Emmanuel and Durairaj (2013)**	Texture	94.7%	250	175	75	5
**Wu et al. (2007)**	Shape, veins	90.0%	1800	1800	320	32
**Kadir et al. (2013b)**	Shape, colour, vein, texture	93.4%	1600	1280	320	32
**Our proposed method**	Margin, lobes, apex, base	94.76%	1907	1280	627	32

The obtained results are outperforming the previous works. This is possibly due to the previous works lack of botanical knowledge. The obtained features of them are unreliable and not worthy. For example for leaf shape, the length, diameter, width and so on of the leaf should not be used as features to recognize the leaf. The age of the leaf may influenced the result. The others work contain features such as the ratio of the venation pixel versus leaf area pixel are considering as unreliable too, as the detected venation using different methods and different magnitude of thresholds provided different answers. By using botanical features, the actual methods to recognize the leaf provided accurate features.

Because the dataset used is a collection of the Acer genus samples leaves from many others dataset, therefore, there are no previous works found on them. However, previous methods are applied on the dataset to compare with the proposed method. The comparison is shown in Table 2. The proposed method still outperformed other previous works as the accuracy achieved is at 82.6 percent. The results show that focusing on apex, base, lobe, and margin provides high accuracy in Acer than Flavia compared to the existing methods. Tables 3 and 4 reveal merits and demerits of some discussed works.

**Table 2:** Comparison of previous proposed method with our proposed method for Acer dataset.

Author	Features	Accuracy	Dataset	Training	Testing	Species
**Wu et al. (2007)**	Shape, veins	37.3%	600	450	150	32
**Kadir et al. (2013b)**	Shape, colour, vein, texture	62.0%	600	450	150	32
**Arun, Emmanuel and Durairaj (2013)**	Texture	43.3%	600	450	150	32
**Our proposed method**	Margin, lobes, apex, base	82.6%	600	450	150	32

**Table 3:** Apex feature extraction.

Authors (year)	Method	Merits	Demerits
Hati and Sajeevan [13], Arun Priya, Balasaravanan and Thanamani [15]	Compute the angle of leaf apex and base.	Very simple Fast Low computation Convenient Can be applied in apex and base	Non-efficient when the leaf is inclined. Different leaf apex type may have same angle. Unable to track the curve of leaf apex and base. Not robust to geometrical transformation.
Pahalawatta [23]	Gradient in sub-window	Simple Can be applied in apex and base	Unable to detect the abruptly changes of leaf apex and base. Not robust to geometrical transformation.
Watchareeruetai, U., Ditthawibun, M., & Phanjan, K. [24]	Symmetry analysis	Simple Low computational	The apex will incline to one side, either right or left or no incline. It will affect the result. Unable to track the curve of leaf apex and base.
Prance, G. T. [25]	Multi-entry key	No program is required. All are manual	Slow Botanist is required to identify the plant species
Kolivand et al.	Proposed method	Able to distinguish the leaf apex and base based on the perspective of botanist. It is important to differentiate what is the type of the apex and base as it is important to record in the patent of plant. To store characteristic state can reduce the usage of space compared to morphometric. However, the morphometric is needed to find the characteristic state. It is challenging to know which is the apex and which is the base if the sample leaf does not have petiole for example the dataset of Flavia and our method able to solve this problem. Some of the leaf have two peaks as their base and apex and some only have one, but our proposed method able to distinguish them.	It is difficult to extract the features based on botanical features. The proposed methods need to find out the regions of apex.

**Table 4:** Margin feature extraction.

Method	Authors (year)	Merits	Demerits
Ripples features	Narayan and Subbarayan [17], Pornpanomchai, Supapattranon and Siriwisesokul [11], Pornpanomchai et al. [19]	Simple approach Able to differentiate the entire leaf (smooth margin without teeth) and teeth leaf.	Unable to differentiate the type of leaf teeth. Damage leaf may affect the result.
Digital morphology	Corney et al. [26], Arun Priya, Balasaravanan and Thanamani [15], Arora et al. [27].	Most of the taxonomist used this method. Easy to compute. Fast.	Same magnitude of angle may had different shape of leaf teeth.
DTW	Cope and Remagnino [28]	Obtained pattern information. Popular.	Memory complexity Slow High computation as this method need to search for optimal time alignment path
margin roughness	Mallah, C., Cope, J., & Orwell, J. [29].	Easy Low computational	Same magnitude of angle may had different shape of leaf teeth.
Kolivand et al.	Proposed method	Able to distinguish the type of the teeth based on the perspective of botanist. It is important to differentiate what is the type of the teeth as it is important to record in the patent of plant. To store characteristic state can reduce the usage of space compared to morphometric. However, the morphometric is needed to find the type of the leaf.	Unable to detect ciliate teeth.

Previous works can give good results to the Flavia dataset which are from higher taxa’s plant. However, when it is from the same genus, the results are not promising. The Acer dataset forms from the plant species of the same genus, therefore, their similarity is very high. The previous works are unable to distinguish them. By embedding the botanical features, the achievement in identifying the plant species is promising.

## Conclusion

In this paper, a new approach is presented to detect the region of a leaf structure. Most of the research in this area focuses on shape, colour, vein, and texture, which consume high levels of computational processing, as can be observed in Tables 1 and 2. There are no more attention on different part of leaves than other parts of a leaf, however, this research focused on phenetic parts of leaf in this regard with a high accuracy. Detecting the local maxima and local minima is completed based on CCD-EBP, using north and south region to recognise the apex and base. Digital morphology is used to measure the leaf shape and the leaf margin. CCG is proposed to extract the curvature of leaf apex and base. Experiments are conducted in both standard datasets of Acer and Flavia. However, we have considered phenetic features and the results are impressive. We believe that by considering some other features such as texture, shape, and venation, we can reach the highest percentage possible utilising these techniques and features.

## Supporting information

S1 Dataset Acer dataset. (PDF)
