# Supplementary material for: An expert botanical feature extraction technique based on phenetic features for identifying plant species
Source: PLoS One. 2018 Feb 8;13(2):e0191447. doi: 10.1371/journal.pone.0191447 (PMC5805256; doi:10.1371/journal.pone.0191447)
Supplement: S1 Dataset — (PDF) [file pone.0191447.s001.pdf]

APPENDIX D

ACER DATASET

Label	Scientific Name	Sample Image	(.jpg)						
1	***Acer Campestre***	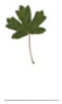 1009.jpg	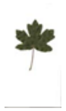 1010.jpg	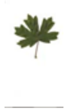 1011.jpg	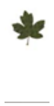 1012.jpg	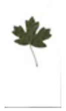 1013.jpg	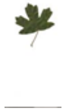 1014.jpg	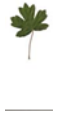 1015.jpg	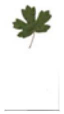 1016.jpg
2	***Acer Ginnala***	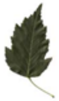 1066.jpg	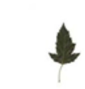 1067.jpg	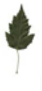 1068.jpg	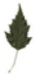 1069.jpg	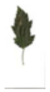 1070.jpg	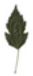 1071.jpg	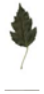 1072.jpg	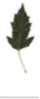 1073.jpg

3	***Acer Negundo***	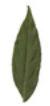 1131.jpg	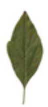 1132.jpg	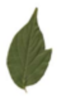 1133.jpg	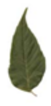 1134.jpg	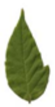 1135.jpg	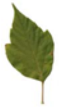 1136.jpg	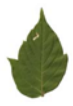 1137.jpg	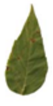 1138.jpg
4	***Acer Palmatum***	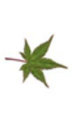 1193.jpg	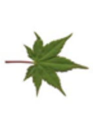 1194.jpg	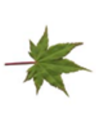 1195.jpg	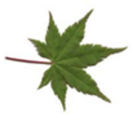 1196.jpg	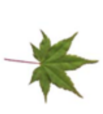 1197.jpg	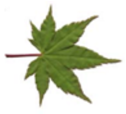 1198.jpg	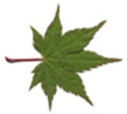 1199.jpg	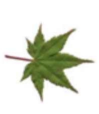 1200.jpg
5	***Acer Platanoides***	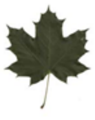 1289.jpg	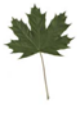 1290.jpg	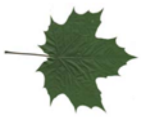 1291.jpg	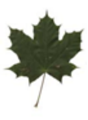 1292.jpg	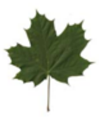 1293.jpg	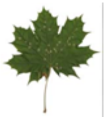 1294.jpg	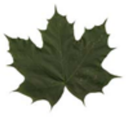 1295.jpg	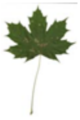 1296.jpg
6	***Acer Pseudoplatanus***	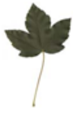 1350.jpg	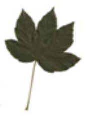 1351.jpg	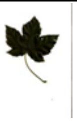 1352.jpg	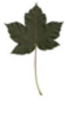 1353.jpg	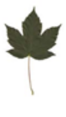 1354.jpg	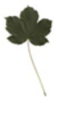 1355.jpg	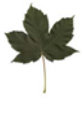 1356.jpg	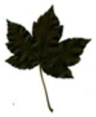 1357.jpg
7	***Acer Saccharinum***	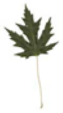 1411.jpg	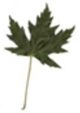 1412.jpg	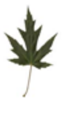 1413.jpg	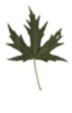 1414.jpg	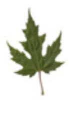 1415.jpg	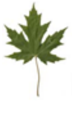 1416.jpg	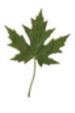 1417.jpg	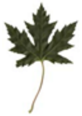 1418.jpg

8	***Acer Saccharum***	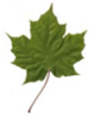 1475.jpg	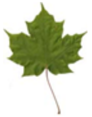 1476.jpg	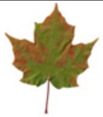 1477.jpg	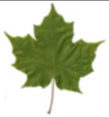 1478.jpg	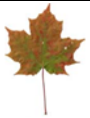 1479.jpg	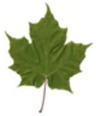 1480.jpg	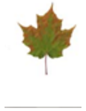 1481.jpg	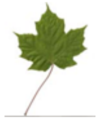 1482.jpg
9	***Acer tataricum***	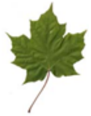 1475.jpg	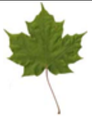 1476.jpg	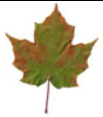 1477.jpg	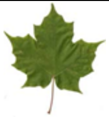 1478.jpg	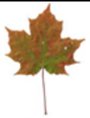 1479.jpg	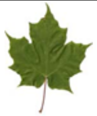 1480.jpg	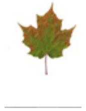 1481.jpg	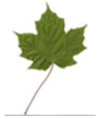 1482.jpg
